# Physiological Impact of Palm Olein or Palm Oil in Infant Formulas: A Review of Clinical Evidence

**DOI:** 10.3390/nu12123676

**Published:** 2020-11-28

**Authors:** Maria Padial-Jaudenes, Esther Castanys-Munoz, Maria Ramirez, John Lasekan

**Affiliations:** 1 Abbott Nutrition R&D, Granada University Science Park, 18016 Granada, Spain; mery.padial@yahoo.es (M.P.-J.); esther.castanysmunoz@abbott.com (E.C.-M.); 2 Abbott Nutrition R&D, Abbott Laboratories, 18004 Granada, Spain; maria.ramirez@abbott.com; 3 Scientific & Medical Affairs, Abbott Nutrition, Abbott Laboratories, Columbus, OH 43219, USA

**Keywords:** palm olein, infant formulas, calcium absorption, fat absorption, DHA absorption, bone mineralization, stool consistency, weight accretion

## Abstract

Palm oil/olein (PO/POL) is used in infant formulas to imitate the fatty acid profile of human milk (HM) and achieve similar levels of palmitic acid (PA). However, the positions of fatty acids on the triacylglyceride differ between PO/POL and HM, which affect fat absorption and produce unintended physiological consequences. Recent papers have reviewed evidence for physiological benefits of PO/POL and beta-palmitate (sn-2-palmitate) in infant formulas. The aim of the present review is to supplement the assessment of available clinical evidence on the physiological effects of PO/POL formulas in healthy infants. We intend to focus on PO/POL and not on sn-2-palmitate, since the latter was recently extensively reviewed. Clinical evidence supports that PO/POL in infant formulas leads to a lower fat, DHA, palmitate and calcium absorption, and bone mineralization; soft stools; and growth (weight accretion) compared to formulas without PO/POL. Consequently, it seems prudent to be considerate and cautious when adding PO/POL to infant formulas. While HM is the gold standard for infant nutrition, the development of infant formula should be based on achieving positive physiological outcomes, rather than just replicating HM nutrient composition.

## 1. Introduction

Infants require optimal nutrition to support normal development and growth. Human milk (HM) is considered as the gold standard for infant nutrition due to its unique composition and delivery of essential nutrients to support optimal growth and development in infants [1]. As a result, infant formulas (IF) are often designed to imitate the composition of HM.

Lipids in HM are an indispensable source of energy and nutrients, such as essential fatty acids (FAs) and phospholipids and are vital to the absorption and metabolism of fat-soluble vitamins and minerals such as calcium, phosphorus, etc., and other liposoluble compounds. They play an important role in meeting the needs of infants to support growth, cognitive and neurological developments [2]. Mature HM has a high content of saturated fatty acids (SFAs, ≈34%–47%), followed by monounsaturated (MUFAs, ≈31%–43%), ω-6 polyunsaturated fatty acids (PUFAs, ≈12%–26%) and ω-3 PUFAs (≈0.8%–3.6%) [3]. In HM, predominant FAs are oleic (OL, C18:1) and palmitic acid (PA, C16:0), which represent approximately 38% and 21% of total FAs, respectively [4].

**Figure 1:**
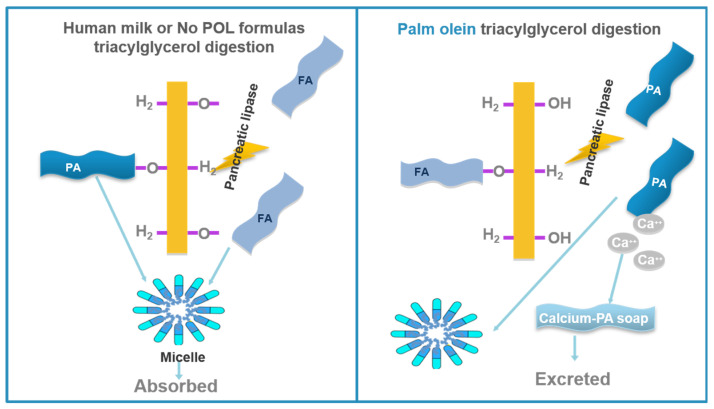
Comparative triacylglyceride digestion in human milk versus palm olein/palm oil based infant formulas. FA = fatty acid; PA = palmitic acid.

In HM and infant formulas, lipids appear predominantly as triacylglycerides (TG). TGs consist of a glycerol molecule backbone and three fatty acids, which can bind to the glycerol molecule in three positions. The positions are named according to a stereospecific numbering (sn) system as sn-1 (alpha), sn-2 (beta) and sn-3, the second being the one in the center. Where each FA is positioned in the TG plays an important role in the way infants absorb fat because of the cleavage specificity of the human pancreatic lipase. This lipase cleaves the sn-1 and sn-3 positions, releasing two free fatty acids (FFAs) and one 2-monoglyceride. Due to their water solubility, the FFAs released from positions sn-1 and sn-3 are better absorbed if they are unsaturated (Figure 1). In contrast, if the FAs situated in sn-1 and sn-3 positions are long chain saturated FAs, such as palmitic (PA) and stearic (SA) acids, they are poorly absorbed when released, and tend to form insoluble calcium soaps. However, when PA is esterified to the sn-2 position, the digestion results in the formation of a water-soluble sn-2 monoglyceride which is well absorbed. In fact, the relative absorption of PA is linearly associated with the proportion located in the sn-2 position of the TG [5]. Even though a high percentage of HM fat is saturated, it is very well absorbed by infants because approximately 70% of the PA is at the sn-2 position on the TG. Thus, the hydrolysis by pancreatic lipase results in the formation of palmitoyl-monoacylglyceride which is water-soluble and well absorbed.

Infant formulas commonly use vegetable oils as a source of fat. Palm olein (POL) is used in the fat blends of infant formulas in order to mimic levels of PA in HM, due to its high content of PA. Contrary to HM, the percentage of sn-2-PA in POL is very low. The high content of sn-1 and sn-3-PA in POL results in lower PA absorption and the consequent formation of calcium-palmitate soaps. These insoluble soaps are excreted in the stools, resulting in a lower absorption of PA and calcium (Figure 1). As a matter of fact, meta-analyses and systematic reviews [6,7] of several clinical studies suggest that infant formulas containing high amounts of PO/POL may have unintended physiologic consequences, such as lower fat and calcium absorption, lower bone mineralization or lower soft stools compared to formulas without PO/POL due to their high content of sn-1,3-palmitate.

The nutritional composition of human milk is an important guide for the design of IF but it is also necessary to consider the physiologic outcomes generated by feeding HM. The ideal approach is to design an IF whose nutritional composition and physiological effects are similar to those observed in healthy infants fed HM.

The aim of the current review is to provide a comprehensive and integrated assessment of the available clinical data on the physiological effects of PO/POL formula intake in healthy infants and to include aspects that will supplement what were covered in previous publications. This review focuses only on PO/POL; not high beta-palmitate (sn-2-palmitate), since the latter has been extensively covered in a recent review paper [8].

## 2. Palm Fruit Derived Oils as a Source of Saturated Fat in Infant Formulas

Palm (*Elaeis guineensis*) is a tree whose fruits are widely used as the source for many types of oils. There are two major types of oils derived from a palm tree; palm oil (PO) is extracted from the mesocarp of the palm fruit and palm kernel oil (PKO) from its nuts [9]. PKO has a higher viscosity and is more saturated than PO since its major fatty acids are myristic and lauric acids. The different fatty acid profiles and distinct physicochemical characteristics of PKO and PO define their industrial use; consequently, PO is mainly used for its nutritional properties and PKO is primarily used in the oleochemical industry [10].

Crude palm oil (CPO) is reddish in color due to its high content of α and β carotenoids which are vitamin A precursors with antioxidant activities. CPO also contains tocotrienols, monoterpenes and polyterpenes. A refining process is needed to remove undesirable compounds in CPO and improve its stability and palatability; consequently, CPO is refined, bleached and deodorized, to obtain a light-colored oil. This process implies the destruction of most carotenoids, but it is the preferred form by most consumers, especially in the western and European countries [11,12].

Two fractions can be obtained from PO, one is solid because it has a high melting point (palm stearin) and the other is liquid due to its low melting point (palm olein). PO and palm olein (POL) are the palm fruit derived oils most commonly used for nutritional purposes. POL and PO are often confused with each other. Despite their common origin, they differ in physical properties, methods of derivations and levels of oleic and palmitic acids (Table 1).

The main differences between PO and POL are related to their fatty acid compositions, physical characteristics and use in food industry. PO contains PA as the major fatty acid (≈44% of total FAs) followed by OL (≈39%) while POL has a higher proportion of OL (≈43%) followed by PA (≈40%). Compared to PO, POL is more liquid at room temperature and its blend of glycerides is more homogeneous. POL is easier to blend with other oils and has a lighter color, making it attractive for food applications and technology. POL is used in many infant formulas to imitate HM as it provides closer ratios of OL and PA, which are the first and second major fatty acids in HM and POL [7,9,13]. It is worth noting that most of the available published clinical studies done in infants evaluated formulas containing POL, but not formulas containing PO. Nonetheless, their physiological effects are thought to be similar.

## 3. Physiological Effects of Palm Olein (POL) or Palm Oil (PO) in Infants

Formulas containing a high proportion of POL in their fat blend have been clinically shown to result in some unintended physiological outcomes in infants which do not seem to be present in infants fed formulas without POL. Several clinical studies support that these formulas may decrease calcium, fat, DHA absorption, reduce bone mineralization and produce harder stools due to their POL content compared to formulas without POL. In addition, some very few clinical studies have demonstrated that POL reduced growth as measured by weight gain in infants. These physiological effects will be discussed under a separate section in this paper.

### 3.1. Calcium and Fat Absorption

It is widely known that calcium plays a critical role in living organisms. Calcium participates in diverse functions, being the main cation among the minerals that form bones. The rate of calcium deposition in bones during infancy is one of the highest at any age, which is similar to the rate noted during adolescence; however, it gradually decreases as growth rate diminishes [14]. Moreover, it is necessary to consider how fat absorption varies when the source of fat in IF changes. A high percentage of the energy in IFs is provided by fat, because fat is the main source of energy during early life and the quality of dietary lipids in infancy is important for physical growth and cognitive development.

Significant data from various published papers [13,15,16,17,18,19] affirm that calcium and fat absorption are lower in infants fed formulas containing POL compared to those fed formulas without POL (Table 2). All the clinical studies highlighted in Table 2 indicated significantly higher fatty acids (palmitic and stearic acids) and calcium soaps in feces of infants fed POL formulas compared to formulas without POL.

A randomized clinical trial (RCT) in preterm infants published only as an abstract by Hansen et al. [19], indicated that calcium and fat absorption had an inverse relationship with the levels of PA from POL in the infant formulas. The study demonstrated a dose dependent lowering of calcium and fat absorption with increasing contribution of PA from POL based formulas, using PA from HM as a reference.

Nelson et al. [17] in a randomized crossover balance study including 11 infants from 27 to 161 days of age, reported a lower fat absorption in every infant fed the formula with POL compared to those fed the formula without POL. Significant differences in average fat absorption between groups (*p* < 0.001) were also reported. Most infants had a lower calcium absorption when POL formula was fed compared with those infants fed the formula with no POL (*p* < 0.01). The study also demonstrated that fecal excretions of fat and calcium were positively correlated. Same authors later conducted another randomized crossover balance study in 10 infants ranging in age from 22 to 192 days [13]. Results of this study were consistent with those obtained in the previous one, and in this case, fat and calcium absorptions were decreased in every single infant fed POL formula compared with those fed formula without POL (*p* < 0.01 and *p* < 0.001, respectively).

Ostrom et al. [18] conducted two separate controlled, randomized, blinded crossover balance studies in normal term infants, comparing two casein hydrolysate formulas (CHF, *n* = 10) and two soy protein formulas (SPF, *n* = 12) with or without POL. The results of the studies confirmed that the inclusion of POL as the predominant fat in infant formulas produces a significant decrease in calcium absorption in infants fed either the CHF or SPF (*p* < 0.01 and *p* < 0.05, respectively), and a significantly lower fat absorption in infants in the CHF study (*p* < 0.01).

In the double-blinded crossover balance RCT by Leite et al. [16], calcium and fat balance and gastrointestinal tolerance were evaluated in 33 healthy term infants. The tolerance study was conducted in 33 infants, *n* = 16–17 per group, and the metabolic balance study was done in 17 infants. They compared two infant formulas, with and without POL and showed that calcium absorption was significantly higher in the formula with POL compared to the formula without POL when calcium intake was not used as a covariate (*p* = 0.023). However, calcium retention was significantly greater with the feeding of formula without POL compared with the formula with POL, with or without calcium intake as a covariate (*p* = 0.024 and *p* = 0.015, respectively). Infants fed the formula without POL had significantly higher fat absorption (*p* = 0.020). In the same study, the absorption of individual fatty acids was analyzed and reported by Souza et al. [15]. The absorption of both arachidonic acid (ARA) and docosahexaenoic acid (DHA) was significantly higher (*n* = 17; *p* = 0.021 and *p* = 0.038, respectively) in infants fed the formula without POL compared to those fed the formula with POL, with or without the use of intake as a covariate in the analyses.

An unpublished pilot study [20] conducted at the University of Oregon, Portland, OR, USA, compared three formulas with different fat blends. One experimental formula contained palm oil (PO) as 60% of the fat blend, while two other formulas serving as control formulas, did not. The two control formulas had corn/coconut fat blend, and sunflower/coconut fat blend, respectively. Sixteen low-birth-weight infants were assigned to one of the three groups (POL, *n* = 9; no POL groups *n* = 4). Data from this study funded by Abbott Nutrition (Columbus, OH, USA) showed that fat and calcium absorption was lower (*p* < 0.05) in infants fed PO formula, compared to infants fed the formulas without PO. Fat absorption was 70.2 ± 4.1 (mean ± SEM), 81.5 ± 2.7 and 80.6 ± 5.3 for PO, corn and sunflower oil groups, respectively. Calcium absorption for PO, corn and sunflower oil groups were 34.2 ± 6.3, 43.5 ± 6 and 41.8 ± 5.3, respectively. Even though this pilot study was not published, it represents the only known study that clinically assessed fat and calcium absorption from palm oil containing formula in infants.

A unique study by Hicks et al. [21] compared fractional calcium absorption in three groups of infants fed either a POL predominant milk-based formula containing prebiotics (POL-PB), one without prebiotics (POL-NoPB) or human milk (HM). The study noted a higher fractional absorption in the HM group (76.0% ± 2.9%; means ± SEM) compared to POL-PB (56.8% ± 2.6%) and POL-NoPB (59.2% ± 2.3%) groups. Despite the absence of a formula without POL in this study, the relevance of the result is evident, as calcium absorption from formulas without POL is closer to that of HM, compared to that from formulas with POL [19]. The addition of prebiotics to the formula with POL in this study was intended to improve calcium absorption since supplemental prebiotics are known to help enhance calcium absorption. The study did not show an improvement with the prebiotics addition. Instead, it showed that both formulas containing POL had lower calcium absorption relative to HM.

The overall conclusion from these clinical studies is that the absence of POL as a source of fat in infant formula improves the absorptions of fat (including DHA and ARA) and calcium, irrespective of whether the formula is based on intact protein, hydrolyzed protein, milk or soy protein and liquid or powder forms. In contrast, the presence of POL in formulas lowers these nutrient absorptions.

### 3.2. Bone Mineralization

Bone mass accretion in early ages is critical to bone health in later life. Inadequate bone mass is related to osteoporosis [22] and childhood fractures [23]. Consequently, optimal bone mass acquisition in infants is an important prevention factor for osteoporosis later in life [24]. Several authors [25,26,27] have demonstrated that bone mineral content (BMC) in infancy is altered if POL formulas are fed, as calcium absorption is impaired.

A double-blind, parallel feeding RCT by Koo et al. [25] evaluated BMC and BMD in 102 healthy infants (formula with POL *n* = 52; formula without POL *n* = 50; Table 3). The study showed that BMC and BMD of infants fed the formula containing POL were significantly lower (*p* < 0.001) than those of the group fed the formula without POL from about 2 weeks of age to 6 months of age. The two formulas compared in this study were the same types of formulas assessed in the Nelson et al. study [13], which reported a lower calcium and fat absorption with the POL based formula. Essentially, the Koo et al. study [25] provided the clinical relevance of the lower calcium absorption noted in the Nelson et al. study [13].

Borschel et al. [26] evaluated bone mineral content (BMC) and density (BMD) in 48 healthy newborn term infants, randomized into two groups of partially hydrolyzed whey protein-based infant formulas (*n* = 24 per group); one fed POL containing formula and the other fed a formula without POL in a blinded parallel feeding RTC. They observed that, at the end of the study (84 days), mean BMC was significantly greater (*p* = 0.0407) in the formula group without POL compared to the group fed formula with POL. However, no significant differences in BMD were found between the groups.

A recently published review paper [8] acknowledged the reducing effects of POL on bone mineralization but remarked that “bone effects seem to be short-lasting.” The review apparently based the remark on two clinical studies [27,28]. One of the studies is a randomized prospective clinical trial by Specker et al. [27], in which they evaluated low, moderate and high mineral (primarily calcium and phosphorus) based IFs. The oil composition of the fat blends was not provided in the paper, but it was later described in the discussion section of the paper by Koo et al. [25]. The low and high mineral formulas were POL based formulas whereas the moderate mineral formula was one without POL. The study was conducted in two phases, during the first and the second 6 months of life in three groups of healthy term infants (*n* = 93). Phase I was interesting to illustrate the influence of POL on BMC as it was explained in Koo et al. [25]. The infants were receiving the feeding regimens from discharge and the first measurement was done at one month. At this point, infants fed formula without POL (*n* = 30) and those fed HM (*n* = 31) had significantly higher BMC (*p* < 0.02) compared to those fed the formula with POL. It was noticed that at one month of age, most of the infants in the HM group were exclusively breast fed. Thereafter the HM group received mix feeding with the PO formula, and the BMC was close to the BMC of the POL formula group. The group with no POL formula had significantly more BMC than the other two groups. Although the difference observed in the BMC in this study cannot be attributed only to the effect of POL, it can also be attributed to the combination of POL and low mineral intake. Nonetheless, it was noteworthy to highlight the shift in the BMC of the HM fed group when supplemented with the POL containing formula. The reason why the bone effect was considered not long lasting is coming from the phase II of the study, in which the infants were randomized to moderate mineral formula, high mineral formula and cow’s milk and there were no differences in BMC at 9 and 12 months. However, it should be noted that after six months of age, IFs are not the only source of nutrition. Infants received complementary feeding, and this additional source of nutrients may somehow diminish the negative effects that POL had in first phase of the study.

The other study is a retrospective study by Young et al. [28], which assessed the effect of feeding formulas with or without POL during the first 4 months of age on BMC and BMD (assessed by dual energy X-ray absorptiometry (DEXA) technology) at 4 years of age. Healthy term infants (*n* = 178) were included in the study, with 65 infants in the formula with POL group, 56 in the formula without POL group and 57 in the HM group. The study concluded that the use of IF containing POL during the first 4 months of life did not impact either BMC or BMD at 4 years of age. The lack of significant difference in this study is likely due to its small sample size. The fact that the BMC of infants fed formula without POL (583 ± 10 g, mean ± SEM) was numerically higher than that of infants fed formula with POL (570 ± 7 g), given the small sample size used in this retrospective study, suggest a high probability of obtaining a significant difference if an appropriate larger sample size was used. A published rebuttal [29] to this study highlighted the weaknesses of the study which included the following characteristics of the study’s methodology (retrospective, possible variability in measurement, no control for potential confounders and underpowered sample size). The rebuttal also stated that the study design could not address the issue on the long-term negative effect of POL formulas on bone mineralization and that a robust, large sample size and long-term feeding study would be needed to address issue.

Based on the totality of the studies reviewed on bone mineralization, there is adequate clinical evidence that formulas with POL reduces BMC in infants. This observation is consistent with the overwhelming clinical data which demonstrated that formulas with POL produces lower calcium absorption compared to formulas without POL. Dietary calcium is a major dietary factor for bone mineralization.

### 3.3. Stool Consistency

Stool consistency is an important parameter to consider because the softness or firmness of stools may affect bowel movements and it may have gastrointestinal (GI) tolerance consequences. When stools are soft, the bowel moves easily. In contrast, when they are too firm it may lead to constipation and infants’ discomfort.

It is well known that diet influences the characteristics of stools, especially stool consistency. Dietary fat influences stool consistency. The long chain saturated FAs (LCSFAs), such as PA and SA, when esterified in sn-1,3 positions, are known to produce hard stool consistency due to their poor absorption and the consequent formation of calcium soaps. It has been demonstrated [30] that stool hardness is directly related to the concentration of calcium soaps of long chain saturated fatty acids. Various clinical studies [16,24,30,31,32] showed that the inclusion of POL as the major oil in the fat blend of infant formulas induced the formation of harder stools compared to the fat blend without POL due to the higher levels of PA in the sn-1 and sn-3 positions of the POL formulas.

Lloyd et al. [31] evaluated changes in stool pattern in two blinded parallel feeding RCT studies (Table 4). The first study assessed 70 healthy term infants who were weaned from exclusive breast feeding to exclusive formula feeding on either a formula with POL (*n* = 35) or a formula without POL (*n* = 35). The results indicated that stools became harder when infants changed from exclusive breast feeding to exclusively formula feeding on the formula with POL compared to the formula without POL. The authors suggested that weaning from breast feeding to formula feeding produces more gradual transition in softer stool consistency on the formula without POL compared with the formula containing POL. In the second study, 65 exclusively formula fed healthy term infants were randomized to formula with (*n* = 33) or without POL (*n* = 32). The study demonstrated that the infants fed the formula with POL had a harder stool consistency versus those fed the formula without POL. Notably, the two studies confirmed that the inclusion of POL in formulas is associated with a significantly greater percentage of harder stools (*p* < 0.01) when fed to infants.

In an open, multicenter non-blinded study, Alarcon et al. [32] compared five groups of healthy term infants (*n* = 6999) who were fed either HM exclusively (*n* = 979), formula with POL exclusively (*n* = 1013), formula without POL exclusively (*n* = 2677), HM plus formula with POL formula (*n* = 635) or HM plus formula without POL (*n* = 1695). Significant differences were reported in stool consistency between the five groups (*p* < 0.001). HM fed infants had the softest stools, followed by those fed the formula without POL. Infants fed the formula with POL had less softer stools. Therefore, the authors of the study concluded that stool characteristics generated by the formula without POL are the most similar to HM while those generated by formula with POL are the least resembling HM.

Borschel et al. [24] enrolled 89 healthy term infants in a double blinded parallel feeding RCT which evaluated stool consistency between infants fed formula with POL (*n* = 40) and those fed formulas without POL (*n* = 410) at 14, 28 and 84 days of age. The formula without POL demonstrated lower mean rank stool consistency (MRSC) compared to the formula with POL throughout the entire study (*p* = 0.0005 at 14 days of age and *p* < 0.0001 from 28 to 84 days of age). Softer stool consistency scores were noted with the feeding of the formula without POL versus the formula with POL. In another blinded RCT by Borschel et al. [32] which assessed 117 healthy term infants fed formulas with or without POL for 4 months, the results obtained were consistent with those of the previous study. Infants fed the formula with POL (*n* = 83) formula produced less softer stool consistency compared to those fed the formula without POL (*n* = 94) during the entire study period (0.0002 ≤ *p* ≤ 0.0114), except at 119 days of age.

Leite et al. [16] enrolled 33 healthy term infants in a blinded crossover balance RCT, including two study periods with a tolerance phase (*n* = 16–17 per group) and a metabolic balance phase each (*n* = 8–9 per group; Table 4). Results from this study showed that MRSC was significantly lower in infants fed the formula without POL compared to those fed the formula with POL during the metabolic phase (*p* < 0.001).

All the studies reviewed consistently reported softer stool consistency in infants fed formulas without POL. This is important since when stools are soft, the bowel moves easily, while when stools are too firm it may lead to constipation and infants’ discomfort.

### 3.4. Growth (Weight Gain)

There are only a few clinical studies that showed a difference in growth between infants fed PO/POL based formulas and those fed formulas without PO/POL. Hansen et al., in their study (only published as an abstract) [34] evaluated growth of term infants fed either cow’s milk based formulas (*n* = 174 females) or soy based formula (*n* = 266 males) with 45% or 60% of total fat as POL for 120 days (Table 5). The study demonstrated a dose-dependent lowering of weight gain per day relative to levels of POL in the formula fat blend. The weight gain per day was significantly lower (*p* < 0.05) at the 60% level compared to the 0 level of POL inclusion, with 2 g/day and 2.4 g/day weight gain reduction for the cow’s milk formula group and the soy formula group, respectively. Because weight gain differences were <3 g/day, the authors concluded that the weight gain differences noted in this study were not clinically meaningful. Nonetheless, the negative impact of POL inclusion in infant formulas on growth becomes more apparent as feeding progressed in 120 days.

A second study noting a difference in weight gain when infants were fed a formula with POL versus a formula without POL was the RTC study by Specker et al. [26]. Although the study primarily focused on BMC and BMD evaluation and mineral content of the formulas, secondary data from this study indicated a numerically higher weight gain in the formula without POL (mean ± SD = 3.42 ± 0.62 kg, *n* = 30) compared to the formula with POL (3.19 ± 0.62, *n* = 30) at 6 months of age. However, the study sample size was not adequately powered as required for a standard growth study in order to detect statistically significant weight gain differences, and the formulas differed on the content of Ca and P: low mineral content (POL formula) and moderate mineral content (no POL formula).

A third pilot study was an unpublished pilot study funded by Abbott Nutrition, Columbus, OH, USA [20] which showed that weight gain of infants fed a formula containing palm oil (PO) was lower compared to infants fed formulas without PO. In this pilot study conducted at the University of Oregon (Portland, OR, USA), fat blends with a 60/40 ratio of palm oil/sunflower, coconut/sunflower, and coconut/corn oil (the two latter ones being controls) were evaluated in cow’s milk-based formulas fed to low-birth-weight infants. The infants fed the PO containing formula had the lowest (*p* < 0.05) weight gain (17 versus 25 g/day versus control), fat absorption (70% versus 82% versus control), and calcium absorption (37% versus 46% versus control) of the 3 study groups. This study seems to be the only available clinical study of infants comparing PO (rather than POL) in infant formula with those without PO.

## 4. Discussion

PO and POL are widely used in the fat blends of IF in order to emulate the levels of PA in HM. Although the fat blend in IF with PO/POL can match the amount of PA present in HM, it fails to match the physiological effects of HM fat in infants. The stereoisomeric structures of the TGs of PO/POL and HM are significantly different. The PA in PO/POL is predominantly located in the sn-1 and sn-3 positions of the TG; whereas, around 70% of PA in HM is at the sn-2 position. The location of PA on the TG molecule of fats has been demonstrated to influence its absorption [5] and other physiological consequences.

Feeding IF with high PA levels similar to those in HM does not seem to offer any advantage, unless the source of fat contains a high percentage of PA in the sn-2 position of the TG [17]. The development of IF should be targeted to achieve optimal physiological outcomes rather than only mimicking the nutritional composition of HM. This is consistent with the recommending statement in a medical position paper by ESPGHAN [35] which stated that “Although the composition of human milk can be a guide to that of infant formulas and breast milk substitutes, gross compositional similarities is not, in itself, an ideal determinant or indicator of the safety and nutritional adequacy of dietary products for infants. A better approach is considered to be the comparison of outcomes in infants fed such products with those seen in healthy infants who have been breast-fed exclusively for 4 to 6 months”. Ideally, it is better to have both compositional and physiological outcome performance benefits that match HM. However, the latter benefit is preferred over just having only the compositional benefit without the outcome performance benefits. Certainly, the formulas without POF provides a closer match to HM’s performance benefits compared to POL formulas.

It is also necessary to consider the fact that PA is not an essential FA. It is found in high amounts in HM and subsequently, in IF that contains PO/POL so as to mimic HM. Yet, the high PA level in IF containing PO/POL does not yield equivalent beneficial physiological outcomes as yielded by HM. In contrast, IF without PO/POL, which are characteristically low in PA (7%–8% PA as % of total fat) have been overwhelmingly shown to produce beneficial physiological outcome benefits similar to those produced by HM [27,31,32] and better than those produced by IFs with PO/POL [6,7]. In essence, the IFs without PO/POL have performance outcome benefits compared to IFs with PO/POL despite the lack of composition (regarding levels of PA) closeness to HM.

Several studies have assessed and identified the negative physiological effects observed when PO/POL are used as the primary source for PA in IFs. It has been demonstrated that PO/POL in IFs leads to a lower absorption of PA [13,17], DHA [15] and total fat [13,16,17,18]. The unabsorbed free PA tends to form calcium fatty acid soaps, resulting in a lower calcium absorption. The unintended consequences of calcium fatty acid soaps are harder stool consistency [7,26,30,31,32,33], higher calcium loss in stools [6,13,17] and lower bone mineralization [6,25,26,27]. A lower bone mineral accretion since a lower bone mass is suggested to be positively associated with osteoporosis later in life and childhood fractures [6,7].

Of the major physiological impact of dietary PO/POL on infants (which includes calcium and fat absorption, bone mineralization and softer stool consistency), growth as indicated by weight gain appears to be the least documented by clinical studies. We have described three studies [20,27,34] that suggested that PO/POL based formulas produce less growth velocity compared to formulas without PO/POL. This lower growth is more apparent in infants with growth deficits as in very low birth weight (VLBW) infants (unpublished Abbott Nutrition study) who need fat as a concentrated source of energy necessary to fuel rapid catch-up growth and to spare protein for tissue accretion. With more formation of calcium fat soaps and consequential lower fat absorption, leading to more fat (energy source) loss in the stools when PO/POL formulas are fed versus formulas without PO/POL. Although, the observation of the effect of PO/POL based formulas on slow weight gain is less apparent in full/normal term healthy infants probably because their energy requirement is not as high as that for the VLBW or preterm infants. Nevertheless, a longer feeding of a PO/POL based formula exclusively in normal term infants may possibly increase the risk of a lower growth rate versus a formula without PO/POL. Such infants should be under medical supervision to monitor their weight accretion.

A recent ESPGHAN’s position paper [8] evaluated infant formulas containing palm oil or beta-palmitate, focusing mainly on the comparison between PO/POL and sn-2 palmitate formulas while limiting the comparison between formulas with PO/POL and formulas without PO/POL. The position paper missed the opportunity to point out that formulas without PO/POL in addition to having a lower level of PA, maybe considered as a viable alternative to formulas with PO/POL. One of the conclusions of the paper states that “There is insufficient evidence to suggest that PO should be avoided as a source of fat in infant formulas for health reasons”. This conclusion, however, did not adequately consider the multiple clinical studies that showed negative health effects of using PO/POL in formulas versus formulas without PO/POL. The position was challenged in a rebuttal [36] published in the same journal, which recommended caution with the addition of PO/POL in IF. In response, the ESPGHAN Committee acknowledged the rebuttal and further stated that more evidence is needed on the possible long-term effects of PO/POL based formula [37].

Although, the various clinical studies highlighted in our current review utilized different study designs (crossover versus parallel feeding; pilot/short-term versus long-term growth), evaluated different formula types with different compositions (intact protein versus hydrolyzed protein; milk protein versus soy protein) and forms (powder versus liquid), and assessed infants of different ages and conditions (preterm versus term infants) and populations (Brazil versus US); the overall negative effects of PO/POL inclusion in infant formulas are similar and consistent. Irrespective of the differences in the multiple studies, a unifying factor is that PO/POL based formula produced lower fat and calcium absorption, bone mineralization [6] and less softer stools [7]. Consequently, the inclusion of PO/POL as a primary source of fat in IFs should be carefully and cautiously evaluated when adding to infant formulas.

## 5. Conclusions

In conclusion, our current review paper provides a comprehensive review of available clinical evidence of the physiological impact of PO/POL in infant formulas. This is intended to update and supplement other reviews currently available in the literature. Our review focused on the effects of formulas without PO/POL versus formulas containing PO/POL rather than focusing on the contrast of sn-2 palmitate (or beta palmitate) to formulas containing PO/POL. We highlighted multiple clinical studies which documented that the consumption of PO/POL predominant formulas compared to that of formulas without POL consistently resulted in lower fat and calcium absorption, bone mineralization and lower soft stool consistency. In addition, we showed that a few studies also demonstrated a reduced growth (weight accretion) in infants fed a PO/POL based formulas, which was not highlighted in previous reviews. The rationale for including PO/POL in some infant formulas is to match the PA levels in HM for compositional benefits. However, the unintended consequences are the loss of the functional outcome benefits. In contrast, formulas that are free of PO/POL have demonstrated functional outcome benefits which are closer to those shown by HM than PO/POL containing formulas. Although more clinical evidence may be needed to elucidate the possible long-term effects of PO/POL formula, it seems plausible to exercise caution when adding PO/POL to infant formulas, especially formulas targeting infants with growth deficit such as VLBW and preterm infants.

## Figures and Tables

**Table 1:** Comparisons of the major palm tree derived oils.

	Palm Kernel Oil (PKO)	Palm Oil (PO)	Palm Olein (POL)
Origin	Kernel	Mesocarp	Palm Oil
Major Fatty acids (% of total Fatty acids)	Lauric acid (≈50%) Myristic acid (≈16%)	Palmitic acid (≈44%) Oleic acid (≈39%)	Oleic acid (≈43%) Palmitic acid (≈40%)
Viscosity	High	Medium	Low

**Table 2:** Characteristics of studies describing fat and calcium absorption effects.

First Author, Year [ref.]	Type of Study	Design of Study	Sample Size			Population Age	Duration	Type of Formula	Intervention Formula	Comparison Formula	Outcomes
			Total	POL	No POL						
Leite et al. 2013 [16] Souza et al. 2017 [15]	DB-RCT crossover	Two periods, each one including a tolerance study feeding two IFs for 14 d, followed by a 4 d metabolic study	33 ↓ 17	16 ↓ 8	17 ↓ 9	68–159 ± 3 days (Term infants)	Tolerance phase: 14 days Metabolic phase: 4 d	Cow’s milk protein-based powder	POL formula containing 44% * POL, 21.7% PKO and 18.5% canola oil	No POL formula containing 41.4% HO sunflower oil, 29.6% coconut oil and 27.6% soy oil	DHA and ARA are better absorbed from the No POL IF [15]. No POL formula is associated with improved absorption of fat [15,16]. Calcium retention is significantly greater when the No POL formula was fed [15,16].
Ostrom et al. 2002 [18]	2 Blinded RCT crossover	Two studies, infants were fed 2 CHFs or 2 SPFs for 7 days, followed by a 3 d balance study	22 10 (CHFs study) 12 (SPFs study)	ND	ND	Mean age: 84 d (POL) 75 d (No POL) 89 d (POL/No POL) (Full-term infants)	Feeding phase: 7 d Balance study: 3 d	CHF: extensively hydrolyzed protein-based. Liquid SPF: soy protein-based liquid	CHF or SPF containing 45% POL, 20% soy, 20% coconut and 15% HO sunflower oils	CHF containing 50% MCT, 40% safflower and 10% soy oils SPF containing 42% HO safflower, 30% coconut and 28% soy oils	POL formulas (CH or SP) were associated with significantly less calcium absorption. CHFs containing POL were associated with significantly less fat absorption.
Nelson et al. 1998 [13]	RCT crossover	Feeding POL or HOS formula for 3 days at least before the 3–4 d metabolic balance study	10	ND	ND	22–192 days (9 term infants, 1 preterm)	Feeding phase: ≥ 3 d Metabolic balance study: 3-4 d	Cow’s milk protein-based liquid	POL formula containing 45% POL, 20% soy, 20% coconut and 15% HO sunflower oils	No POL formula containing 42% HO safflower, 30% coconut and 28% soy oils	In every single infant: - Fat absorption was significantly less when POL formula was fed. - Calcium absorption decreased significantly when POL formula was fed.
Nelson et al. 1996 [17]	RCT crossover	Feeding POL or No POL formula for 7 days at least before a balance study	11	ND	ND	27–137 days (Term infants)	Feeding phase: ≥ 7 d Metabolic balance study: 3–4 d	Cow’s milk protein-based liquid	POL formula containing 53% POL and 47% soy oil	No POL formula containing 60% soy oil and 40% coconut oil	In every single infant absorption of fat was significantly less with POL formula. In most infants, the percentage of calcium absorption was significantly lower.
Hansen et al. 1996 [19]	RCT	Infants were fed 3 IFs with different % of PA for 7 days before a 3 d balance study	30	ND	ND	ND (Preterm infants)	Feeding phase: 7 d Balance study: 3 d	ND	3 POL formulas containing 10%, 22% and 27% of PA each	Human milk	Fat and Ca absorption are inversely (not linearly) related to PA content. An IF with appropriate Ca content results in fat an Ca absorption similar to HM regardless of PA position in TG
Abbott Nutrition 1982 [20]	Clinical study	Infants were fed a PO experimental IF or one of two control formulas without PO. A 96 h metabolic balance study was carried out	16	9	4 (Coconut/Corn) 3 (Coconut/Sunflower)	5 days (low-birth-weight infants)	Feeding phase: 3 d (full feeds) Metabolic balance study: 96 h	Cow’s milk protein-based powder	PO formula containing 60% PO and 40% sunflower oil	No PO formula containing 60% coconut and 40% corn oils No PO formula containing 60% coconut and 40% sunflower oils	The PO formula provides a poorly absorbed source of fat for low-birth-weight infants, and it is significantly lower than those provided by PO free formulas. The lower mean calcium absorption associated to the PO formula may be related to de poor absorption of fat.

ARA: arachidonic acid; CH: casein hydrolysate; CHF: casein hydrolysate formula; CM: cow milk; DB-RCT: double blinded randomized clinical trial; DHA: docosahexaenoic acid; HM: human milk; HO: high oleic; HOS: high oleic safflower oil; IF: infant formula; MCT: medium chain triglycerides; ND: not determined; No POL: palm olein free; PHF: partially hydrolyzed formula; PKO: Palm kernel oil; POL: palm olein; RCT: randomized clinical trial; SP: soy protein; SPF: soy protein formula; * % indicates percentage of total fats; N (recruited) → N (completed the study).

**Table 3:** Characteristics of studies describing bone mineralization effects.

First Author, Year [Ref.]	Type of Study	Design of Study	Sample Size			Population Age	Duration	Type of Formula	Intervention Formula	Comparison Formula	Outcomes
			Total	POL	No POL						
Borschel et al. 2012 [26]	DB-RCT multicentre	Infants were randomly assigned to either POL or No POL formula during 84 days of life.	89 ↓ 48	44 ↓ 24	45 ↓ 24	0–8 days (Term infants)	84 days	Partially hydrolyzed whey -based Powder	PHF containing 46% POL, 26% soy oil, 20% coconut oil and 6% HO safflower or sunflower oil	PHF containing 41% HO safflower, 29% soy and 27% coconut oils	BMC is significantly greater in the No POL formula, only when body weight is used as a covariate.
Young et al. 2005 [28]	Retrospective	Children fed exclusively HM or IF with or without POL during the 4 first months were recruited. BMC was analyzed by DEXA at 4 years.	178	65	56 57 (HM)	4,5 years	NA	Milk protein-based	IF containing 45% POL, 20% soy oil, 20% coconut oil and 15% HO sunflower oil.	IF containing 40% HO safflower oil, 30% soy oil and 30% coconut oil.	Feeding infants with the PO formula during the first 4 months of life does not have a negative effect on BMC and BMD at 4 years of age. Challenged by Koo [29].
Koo et al. 2003 [25]	DB-RCT	Two groups of infants were fed either PO or No POL formula for the first 6 months	128 ↓ 102	63 ↓ 52	65 ↓ 50	6 ± 1 days (Term infants)	6 months	Cow’s milk protein-based	IF containing 45% POL, 20% soy oil, 20% coconut oil and 15% HO sunflower oil	IF containing 40% HO safflower oil, 30% soy oil and 30% coconut oil	The inclusion of PO in IF at levels needed to provide a fatty acid profile similar to that of HM leads to lower bone mineralization.
Specker et al. 1997 [27]	Randomized prospective Phase I	Infants were randomized (first 6 months) to a low (POL) or moderate (No POL) mineral formula. Another group was BF, supplemented with low mineral IF.	101 ↓ 92	30 (Low) 31 (HM + Low)	31 (Mod)	<14 days (Full-term infants)	6 months	Cow’s milk protein-based	Low mineral IF containing POL and 430 mg/L of Ca	Moderate mineral IF not containing POL and containing 510 mg/L of Ca	The low mineral formula and the human milk fed groups had similar BMC, which was lower than that in the moderate mineral group at 3 and 6 months of age.
	Randomized prospective Phase II	The same infants were randomized (6 to 12 months of age) again to a moderate (No POL) or high (POL) mineral formula or to whole cow’s milk (CM).	92 ↓ 87	39 (High)	38 (Mod) 10 (CM)	<7 months (Full-term infants)	6 months	Cow’s milk protein-based	High mineral IF containing POL and 1350 mg/L of Ca.	Moderate mineral IF not containing POL and containing 510 mg/L of Ca	No significant differences were found in BMC between feeding groups either at 9 or 12 months of age. The effect of mineral intake and fat composition on BM accretion seems not to be long term.

BF: breastfed; BMC: bone mineral content; CM: cow’s milk; DB-RCT: double blinded randomized clinical trial; HM: human milk; HO: high oleic; IF: infant formula; No POL: palm olein free; PHF: partially hydrolyzed formula; POL: palm olein; DEXA: dual energy x-ray absorptiometry; N (recruited) → N (completed the study).

**Table 4:** Characteristics of studies describing stool consistency effects.

First Author, Year [Ref.]	Type of Study	Design of Study	Sample Size			Population Age	Duration	Type of Formula	Intervention Formula	Comparison Formula	Outcomes
			Total	POL	No POL						
Borschel et al. 2014 [33]	Blinded RCT multicentre	Infants were randomized to 1 of 2 PHFs (POL/No POL) during the first 4 months of life.	209 ↓ 177	101 ↓ 83	108 ↓ 94	0–8 days (Term infants)	119 days (4 months)	Partially hydrolyzed whey -based (with added prebiotic GOS) Powder	PHF containing 46% POL, 26% soy oil, 20% coconut oil, 6% HO safflower or sunflower oil	PHF containing 41% HO safflower, 29% soy and 27% coconut oils.	MRSC is significantly lower when PHFs without POL are fed.
Leite et al. 2013 [16]	DB-RCT crossover	Two periods, each one including a tolerance study feeding two formulas for 14 d, followed by a 4-d metabolic study	33 ↓ 17	16 ↓ 8	17 ↓ 9	68–159 days (Term infants)	Tolerance phase: 14 d Metabolic phase: 4 d	Cow’s milk protein-based Powder	POL formula containing 44% POL, 21,7% PKO and 18,5% canola oil	No POL formula containing 41,4% HO sunflower oil, 29,6% coconut oil and 27,6% soy oil	Infants fed No POL formula had significantly softer stools (3.0 + 0.5; mean ± SD) than those fed POL formula (2.4 ± 0.3). MRSC score: 5 = watery, 4 = loose/mushy, 3 = soft, 2 = formed, 1 = hard (higher is softer)
Borschel et al. 2012 [26]	DB-RCT multicenter	Infants were randomly assigned to either a POL or No POL formula during 56 and 84 days of life.	89 ↓ 75 ↓ 64	44 ↓ 37 ↓ 26	45 ↓ 38 ↓ 28	0–8 days (Term infants)	56 and 84 days 56 days ↓ 84 days	Partially hydrolyzed whey -based Powder	PHF containing 46% POL, 26% soy oil, 20% coconut oil, 6% HO safflower or sunflower oil	PHF containing 41% HO safflower, 29% soy and 27% coconut oils	MRSC is significantly greater in infants fed PHF with POL.
Alarcon et al. 2002 [32]	Open, not blinded multicenter controlled	The study was conducted in 17 countries. Infants were fed 1 of 5 diets: HM, No POL, POL, HM + No POL or HM + POL for 14 days.	7673 ↓ 6999	1013 (POL) 635 (HM + POL)	2677 (No POL) 979 (HM) 1695 (HM + No POL)	28–98 days (Term infants)	14 days	Cow’s milk protein-based	POL formula containing 45% POL, 20% coconut, 20% soy and 15% sunflower oils	No POL formula containing 42% HO safflower oil, 30% coconut oil and 28% soy oil. (This composition may vary by country).	Stools of infants fed No POL formula are significantly softer than those of infants fed POL containing formula.
Lloyd et al. 1999 [31] *Study 1*	Blinded RCT	Exclusively breastfed infants were randomized to either formula with or without POL.	82 ↓ 70	39 ↓ 35	43 ↓ 35	4–188 d (No POL) 8–181 d (POL) (Term infants)	BF phase: 3 d Weaning period: ≈ 30 d Exclusively FF period: 14 d	Cow’s milk protein-based	Cow milk based IF containing 45% POL, 20% coconut, 20% soy and 15% HO sunflower oils	Cow milk based IF containing 42% HO safflower, 30% coconut and 28% soy oils	The stools became firmer as infants moved from breastfed to weaning to exclusively formula feeding. Infants fed POL formula had significantly firmer stools in both periods (weaning and exclusively FF)
Lloyd et al. 1999 [31] *Study 2*	Blinded RCT	Exclusively formula fed infants were randomized to either formula with or without POL.	87 ↓ 65	42 ↓ 33	45 ↓ 32	12–16 d (No POL) 12–17 d (POL) (Term infants)	Standard IF feeding phase: 7 d Exclusively FF period: 14 d	Cow’s milk protein-based	Cow milk based IF containing 45% POL, 20% coconut, 20% soy and 15% HO sunflower oils	Cow milk based IF containing 42% HO safflower, 30% coconut and 28% soy oils	Infants fed POL formula had significantly higher average stool consistency.

BF: breastfed; DB-RCT: double blinded randomized clinical trial; FF: formula fed; HM: human milk; HO: high oleic; IF: infant formula; MRSC: mean ranking stool consistency; No POL: palm olein free; PHF: partially hydrolyzed formula; POL: palm olein; RCT: randomized clinical trial; N (recruited) → N (completed the study).

**Table 5:** Characteristics of studies describing growth and weight gain effects.

First Author, Year [Ref.]	Type of Study	Design of Study	Sample Size			Population Age	Duration	Type of Formula	Intervention Formula	Comparison Formula	Outcomes
			Total	POL	No POL						
Specker et al. 1997 [27]	Randomized prospective *Phase I*	Infants were randomized (first 6 months) to a low (POL) or moderate (No POL) mineral formula. Another group was BF, supplemented with low mineral IF.	101 ↓ 92	30 (Low) 31 (HM + Low)	31 (Mod)	<14 days (Full-term infants)	6 months	Cow’s milk protein-based	Low mineral IF containing POL and 430 mg/L of Ca	Moderate mineral IF not containing POL and containing 510 mg/L of Ca	The low mineral formula and the human milk fed groups had significantly less weight gain that moderate mineral formula fed group at 6 months of age.
	Randomized prospective *Phase II*	These infants were randomized (6 to 12 months of age) again to a moderate (No POL) or high (POL) mineral IF or to whole CM.	92 ↓ 87	39 (High)	38 (Mod) 10 (CM)	<7 months (Full-term infants)	6 months	Cow’s milk protein-based	High mineral IF containing POL and 1350 mg/L of Ca.	Moderate mineral IF not containing POL and containing 510 mg/L of Ca	No significant differences were found in weight gain between feeding groups at 12 months of age. The effect of mineral intake and fat composition on weight gain seems to be short term.
Hansen et al. 1996 [34]	RCT	Infants were fed 3 formulas with different % of PA for 7 days before a 3-d balance study	440 174 (CM) ↓ 131 266 (Soy) ↓ 165	40 (45%) 46 (60%) 55 (45%) 46 (60%)	45 64	ND (Term infants)	120 days	ND	Cow milk or Soy based formulas containing 45% or 60% of POL	Cow milk or Soy based formula not containing POL	Differences in growth between 0% and 60% POL formulas are statistically significant but not clinically significant (< 3 g/d). Using a formula with 45% POL to achieve a FA profile similar to HM results in growth equivalent to standard formulas without POL.
Abbott Nutrition 1982 [20]	Clinical study	Infants were fed a PO experimental IF or one of two control formulas without PO. Mean weight gain was analyzed	16	9	4 (Coconut/Corn) 3 (Coconut/Sunflower)	5 days (Low-birth-weight infants)	7 days	Cow’s milk protein-based Powder	PO formula containing 60% PO and 40% sunflower oil	No PO formula containing 60% coconut and 40% corn oils No PO formula containing 60% coconut and 40% sunflower oils	The infants that were fed the PO formula had the lowest (*p* < 0.05) weight gain of the 3 study groups.
Borschel et al. 2014 [33]	Blinded RCT multicenter	Infants were randomized to 1 of 2 PHFs (POL/No POL) during the first 4 months of life.	209 ↓ 177	101 ↓ 83	108 ↓ 94	0–8 days (Term infants)	119 days (4 months)	Partially hydrolyzed whey -based (with added prebiotic GOS) Powder	PHF containing 46% POL, 26% soy oil, 20% coconut oil and 6% HO safflower or sunflower oil	PHF containing 41% HO safflower, 29% soy and 27% coconut oils.	No significant differences in growth or weight gain were found between groups.
Borschel et al. 2012 [26]	DB-RCT multicenter	Infants were randomly assigned to either POL or No POL formula during 84 days of life.	89 ↓ 64	44 ↓ 26	45 ↓ 28	0–8 days (Term infants)	84 days	Partially hydrolyzed whey -based Powder	PHF containing 46% POL, 26% soy oil, 20% coconut oil and 6% HO safflower or sunflower oil	PHF containing 41% HO safflower, 29% soy and 27% coconut oils.	No significant differences in growth or weight gain were found between groups.
Koo et al. 2003 [25]	DB-RCT	Two groups of infants were fed either POL or No POL formula for the first 6 months	128 ↓ 102	63 ↓ 52	65 ↓ 50	6 ± 1 days (Term infants)	6 months	Cow’s milk protein-based	IF containing 45% POL, 20% soy oil, 20% coconut oil and 15% HO sunflower oil	IF containing 40% HO safflower oil, 30% soy oil and 30% coconut oil	There was no significant difference between study groups in weight, length or head circumference over the course of the study.
Lloyd et al. 1999 [31] *Study 1*	Blinded RCT	Exclusively breastfed infants were randomized to either formula with or without POL.	82 ↓ 70	39 ↓ 35	43 ↓ 35	4–188 d (No POL) 8–181 d (POL) (Term infants)	BF phase: 3 d Weaning period: ≈ 30 d Exclusively FF period: 14 d	Cow’s milk protein-based	Cow milk based IF containing 45% POL, 20% coconut, 20% soy and 15% HO sunflower oils	Cow milk based IF containing 42% HO safflower, 30% coconut and 28% soy oils	There were no significant differences in weight gain between feeding groups.
Lloyd et al. 1999 [31] *Study 2*	Blinded RCT	Exclusively formula fed infants were randomized to either formula with or without POL.	87 ↓ 65	42 ↓ 33	45 ↓ 32	12–16 d (No POL) 12–17 d (POL) (Term infants)	Standard IF feeding phase: 7 d Exclusively FF period: 14 d	Cow’s milk protein-based	Cow milk based IF containing 45% POL, 20% coconut, 20% soy and 15% HO sunflower oils	Cow milk based IF containing 42% HO safflower, 30% coconut and 28% soy oils	There were no significant differences in weight gain between feeding groups.

BF: breastfed; CM: cow’s milk; DB-RCT: double blinded randomized clinical trial; FA: Fatty acid; GOS: Galacto-oligosaccharides; HM: human milk; HO: high oleic; IF: infant formula; ND: Not determined; No POL: palm olein free; PA: Palmitic acid; PHF: partially hydrolyzed formula; PO: Palm oil; POL: palm olein; N (recruited) → N (completed the study).
